# Distinguishing Fowler’s and Semi-Fowler’s Patient Postures Within Continuous-Wave Functional Near-Infrared Spectroscopy During Auditory Stimulus and Resting State

**DOI:** 10.3390/brainsci15111172

**Published:** 2025-10-30

**Authors:** Seth Bolton Crawford, Daniel X. Liu, Caroline Joyce Caveness, Rachel Eimen, Audrey K. Bowden

**Affiliations:** 1 Vanderbilt Biophotonics Center, Vanderbilt University, Nashville, TN 37232, USA; seth.b.crawford@vanderbilt.edu (S.B.C.); daniel.x.liu@vanderbilt.edu (D.X.L.); caroline.j.caveness@vanderbilt.edu (C.J.C.); rachel.l.eimen@vanderbilt.edu (R.E.); 2 Department of Biomedical Engineering, Vanderbilt University, Nashville, TN 37232, USA; 3 Department of Electrical and Computer Engineering, Vanderbilt University, Nashville, TN 37232, USA

**Keywords:** fNIRS, spectroscopy, posture, Fowler’s, semi-Fowler’s, standing, sitting, supine, naturalistic, healthcare

## Abstract

**Background/Objectives**: Lightweight and portable functional near-infrared spectroscopy (fNIRS) systems enable neuromonitoring in clinical environments such as operating rooms. Patient posture is known to influence physiology, behavior, and brain activity, and may affect fNIRS measurements. However, the effects of some postures commonly used in clinical care—such as Fowler’s and semi-Fowler’s—remain largely unexamined in fNIRS research. **Methods**: We conducted a singular study in a mock operating room exploring the effects of five postures—standing, upright sitting, Fowler’s, semi-Fowler’s, and supine—on fNIRS data during resting-state conditions and under various auditory stimuli. We collected hemodynamic data and extracted the characteristic hemodynamic response function (HRF) at each posture in response to the presented auditory stimulus and the amplitude of the resting-state signal. **Results**: For the auditory task condition, we found that posture had no statistically significant impact on the amplitude of the global HRF for Fowler’s and semi-Fowler’s postures. We also found no significant relationships across different postures when analyzing the amplitude of the global resting-state signal; however, binning of frequency-dependent postural effects revealed statistically significant differences between Fowler’s and semi-Fowler’s postures at low frequencies (f < 0.09 Hz). **Conclusions**: Our results suggest posture effects need not require complex data processing pipelines or data segmentation efforts on an auditory task-induced condition or on the general analysis of the global resting signal; however, not all reclined postures are equivalent, and we recommend that researchers report the angle of reclination measurements for seated data collection sessions for improved reliability and data context.

## 1. Introduction

**Figure 1:**
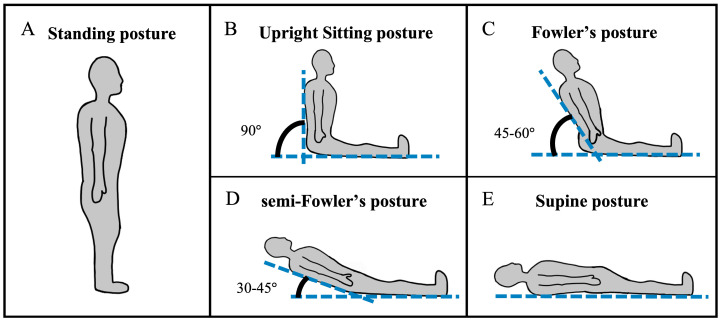
The postures assumed in this study include (**A**) standing, (**B**) upright sitting, (**C**) Fowler’s, (**D**) semi-Fowler’s, and (**E**) supine postures.

Continuous-wave functional near-infrared spectroscopy (cw-fNIRS) is a light-based method for neuromonitoring that can be used to estimate changes in cerebral metabolic activity from multiple brain regions concurrently [1,2,3]. Cw-fNIRS systems are non-invasive and have high potential to be made lightweight and portable. These characteristics facilitate their deployment in a variety of environments outside of typical lab settings, such as in healthcare settings like intensive care units and operating rooms [4]. Yet neuromonitoring in healthcare settings presents challenges for researchers and requires shifts in data collection procedures compared to standard lab experiments. For example, while routine cw-fNIRS experiments require participants to assume a sitting posture compatible with cognitive testing, patients in healthcare settings assume a variety of postures, such as supine, standing, upright sitting, and reclined sitting (Figure 1), throughout the clinical workflow. Importantly, previous studies across numerous imaging modalities have shown that heart rate, blood flow, respiratory volume, oxygen consumption, body temperature, hormone secretion, and other indicators of physiological arousal are posture-dependent [5,6,7,8,9]. Specifically, recent studies utilizing transcranial Doppler (TCD) ultrasound [10], cerebral oxygenation [11], magnetic resonance imaging (MRI) [12,13,14,15], or a combination of these modalities [16] have shown that hemodynamic signals are affected by participant posture or the transition between postures. Given that the cw-fNIRS source signal is hemodynamic in nature, it is expected that posture will have an effect on some aspect of the cw-fNIRS signal in the resting state, and the failure to account for posture-related effects may impair analysis. Hence, a primary goal of this work is to report the effects of healthcare-relevant postures on cw-fNIRS hemodynamic data so that newer, portable systems can be accurately and reliably applied in the clinic.

Some studies on the effects of posture on fNIRS data already exist. For example, a study by Almulla et al. collected and compared fNIRS data from the primary motor cortex as patients moved between upright sitting and standing positions. The study showed a significant increase in the task-induced oxygenated hemoglobin (HbO) concentration when participants moved from standing to sitting, compared to moving from sitting to standing [17]. Almulla posited that the significant change was most likely rooted in physiological or systemic effects associated with the transition in posture and was less likely a result of increased motor activation.

Similarly, Ozgoren et al. investigated the effects of switching between postures. Their work expanded the range of tested postures to include the transition to lying supine in addition to standing and sitting upright [18]. Ozgoren collected data across the prefrontal cortex and reported that during the transition, or the task-induced state, the deoxygenated hemoglobin concentration (HbR) was the least affected fNIRS recording, and the change in HbO concentration increased significantly when transitioning from sitting to supine. Ozgoren also discussed the change in HbO concentration as being physiologically induced, as opposed to motor-induced, and transient, with the signals recovering after four minutes.

Together, the Almulla and Ozgoren works show a consistent trend that the task-induced HbO concentration when switching between postures generally increases as the posture transitions to a greater angle of reclination. This trend can also be supplemented by recent work from Kim et al. showing the effects of dynamic postural transitions on cerebrovascular regulation, which was extracted from the low-frequency hemodynamic oscillations measured with fNIRS [19]. However, all three of these studies focus only on the transition and do not investigate how hemodynamic data change when collected at different postures under a single task-induced or resting state. Understanding the isolated effects of individual postures on fNIRS data is vital for ensuring comparability and interpretation of data during studies where participants remain still in a single posture.

More recent postural studies in the fNIRS field have investigated how measured hemodynamics are impacted by distinct postures, including those that can be assumed outside of the lab environment. For example, work by Li et al. leveraged a portable cw-fNIRS system during standing, balancing tasks [20]. Similarly, a study by Qi et al. used fNIRS during a Tai Chi standing meditation [21]. Both of these works showed changes in cortical hemodynamic patterns in the different standing postures assumed; however, their applicability to understand effects on other, seated postures that may be assumed outside of the lab environment is limited.

A study by Chen et al. focused on the effects of individual postures and investigated the relationship between the fNIRS global resting-state signal and electroencephalography (EEG) vigilance [9]. In the study, participants assumed supine, sitting, and standing postures during data collection, and the task-induced hemodynamic response function (HRF) amplitude to an auditory stimulus was used as a control because it was initially hypothesized, and later proven by Chen et al., that it did not differ across postures. However, Chen reported a significant increase in HbO signal amplitude during the resting-state condition at a frequency range above 0.05 Hz in standing and sitting postures compared to the supine posture. These findings demonstrate that posture can significantly influence resting-state fNIRS signals when viewed within a particular frequency window. This may thus be relevant to data collected in healthcare settings, where patients may experience prolonged resting states in a single posture.

**Figure 2:**
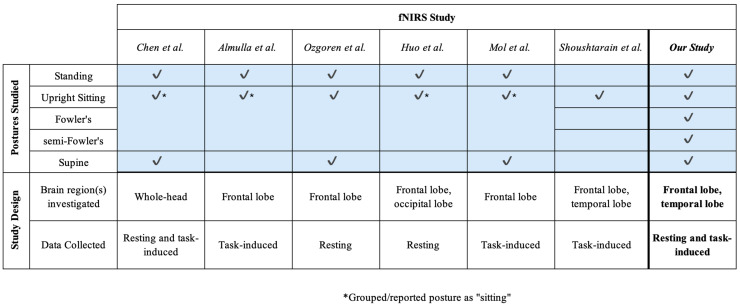
An overview of fNIRS-based studies investigating postural effects that also informed the design of this study [9,17,18,22,23,25]. Check marks indicate which postures were assumed during each study.

Although a number of these prior works (Figure 2) cumulatively investigate different postures, the range of postures explored by any one study was limited. Importantly, the postures tested in these studies did not include Fowler’s and semi-Fowler’s postures but instead focused on a single “sitting” group [9,17,18,22,23]. Fowler’s and semi-Fowler’s postures fall under the reclined sitting label but are unique with varying levels of reclination [9]. These two distinct postures are among the most commonly assumed in healthcare settings, as they are optimal for physiological processes such as improved oxygenation, homeostasis, and access to the chest cavity during surgery [7,8,24]. Fowler’s posture aids in the digestion process and is commonly assumed when patients are eating, swallowing, and defecating, while semi-Fowler’s is used to administer drugs and is considered the ideal position for patients to rest and recover. While there is a relatively small contrast between these two postures in terms of reclination angle, the difference can impart large changes in terms of venous return and cerebral perfusion, and influence measured hemodynamic signals [5,7,8]. Understanding the effects of both Fowler’s and semi-Fowler’s postures will ensure that cw-fNIRS systems can yield accurate and reliable data when utilized in clinical environments; therefore, these two postures and their effects on data should be investigated individually.

In an attempt to conduct a singular study on the effect of postures adopted in healthcare settings during prolonged periods of static posture on cw-fNIRS data, we tested the effects of five postures on the amplitude of the HRF in response to an auditory stimulus and on the global resting-state signal, defined as the standard deviation of the global resting time series. Our study design is an extension of the work previously conducted by Chen et al. [9] with the investigation of additional postures; the postures we considered in the study included Fowler’s and semi-Fowler’s postures in addition to standing, sitting, and supine. Importantly, all five postures were tested on the same subject over multiple data collection periods, which helps provide a more unified picture of the response than when one must look across multiple studies.

Based on Chen’s previously published findings of significant decreases in the amplitude of the global resting-state signal when comparing standing to sitting and supine postures, we hypothesized that the amplitude of the global resting-state signal would decrease as the angle of reclination for the tested postures decreases (i.e., the fluctuations or variability in the signal produced under semi-Fowler’s and supine postures would be less than those produced under upright sitting and Fowler’s postures). In addition to being rooted in Chen’s findings, this hypothesis is also grounded in expectations of how known physiological factors that were discussed earlier in this work—decreased physiological arousal, increased stability of heart rate, blood pressure, and cerebral autoregulation, and decreased respiratory rate and oxygen consumption when in more reclined postures—will likely lead to less variability in the recorded resting-state hemodynamic signals [5].

Our work marks the first study of the effects across standing, upright sitting, Fowler’s, semi-Fowler’s, and supine postures on cw-fNIRS data. The novel results around statistically relevant differences between Fowler’s and semi-Fowler’s posture on global resting-state data can be used to improve the accuracy of neuroimaging results collected in relevant healthcare settings and other settings outside of the typical lab environment.

## 2. Materials and Methods

### 2.1. Recruitment

We recruited *n* = 15 healthy participants (mean age 28.5 yrs, 7M/8F) to undergo an auditory task during the collection of cw-fNIRS data. Participants included in the study had no reported history of neurological or hearing disorders. The experimental procedure was approved by the Vanderbilt University Institutional Review Board (Vanderbilt IRB#231350).

### 2.2. Auditory Stimulus

While our primary analytical focus was planned for resting-state data, our study design also included a task condition. Similarly to the work by Chen et al. [9], we incorporated the task condition to act as our study control; further, we aimed to use the task condition as a signal-quality and data-accuracy check by ensuring a consistent functional response was recorded and that the proper functional regions were engaged during the stimulus condition. Our task condition was designed to activate the auditory cortex in the temporal gyrus of the brain. The auditory cortex is an ideal region to study posture effects because auditory-based cw-fNIRS procedures are known to be reliable on the group level [26] and have been used in other studies of posture effects [27,28]. Further, previous work by Fu et al. has proven that auditory stimuli are perceived in patients under general anesthesia, and the auditory-evoked response can be used to measure the depth of anesthesia [29,30,31]; these specific applications of auditory stimuli demonstrate their usefulness in studies extending to healthcare settings.

To create the auditory stimulus, we utilized audio files from the International Collegium of Rehabilitative Audiology (ICRA) as the auditory stimuli during the procedure [32]. ICRA noise consists of signals with speech-like spectral and temporal properties but is not understandable as speech. ICRA noise is a well-known and accessible stimulus that has been used in numerous auditory studies utilizing fNIRS and fMRI [33,34,35,36,37,38]. The stimulus was delivered binaurally via earbud headphones (Kurdene—Shenzhen, China), which were chosen for stimulus delivery because their wireless design does not interfere with the cw-fNIRS cap or electronics setup. Additionally, the headphones have noise-canceling capabilities that refine our control over the stimulus conditions we applied during the study.

### 2.3. cw-fNIRS System

**Figure 3:**
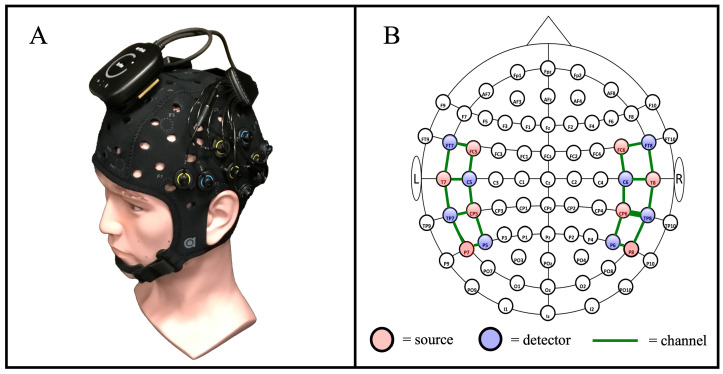
An overview of the cw-fNIRS system used for the auditory stimulus procedure. (**A**) The commercial Artinis Brite MKII system was used for data collection. (**B**) The layout of system sources and detectors, in alignment with the 10-10 international layout [39], was used to measure the hemodynamic response across the temporal region of the brain [40].

Data collection was accomplished using a commercial, cw-fNIRS Brite MKII system (Artinis Medical Systems—Gelderland, The Netherlands) operating at wavelengths of 760 and 850 nm and collecting data at a rate of 50 Hz. The Brite MKII system is lightweight, portable, and can transmit data wirelessly, providing ample freedom of movement during the study. The source and detector probes were configured to have a source-detector separation distance of 30 mm, which is typical for probing the cortical level of the adult brain, and they were positioned to cover primarily the temporal gyrus, including the auditory cortex, and the inferior prefrontal gyrus in accordance with the 10-10 international optode standard cap-based sensor layout compatible with EEG and fNIRS [39]. An overview of the cw-fNIRS system is provided in Figure 3, which includes an image of the Artinis Brite MKII system (Figure 3A) and a diagram showing the layout of the sources and detectors (Figure 3B). We used a 22-channel configuration—20 long-separation channels and 2 short-separation channels—consisting of eight sources and eight detectors arranged to cover the inferior frontal cortex and temporal lobe [40].

### 2.4. Posture Conditions

For each participant, we recorded the hemodynamic response (both HbO and HbR signals) to the auditory stimuli at each of the five postures: standing, upright sitting, Fowler’s, semi-Fowler’s, and supine. To achieve the standing position, participants stood vertically, creating a 90° angle with the floor, with the cw-fNIRS device and headphones in place without any furniture on which to balance or lean. The participants were asked to remain balanced across both feet without any swaying or shifting of weight and without moving their heads.

**Figure 4:**
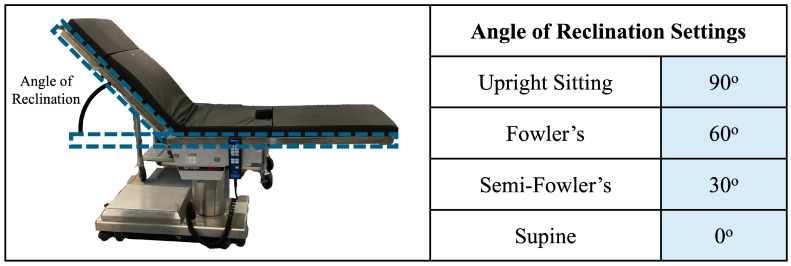
Overview of posture determination. The angle of reclination was determined with the help of bars attached to the surgical table, which are outlined in blue dashed boxes. The surgical table was ready for experimentation when the angle matched one of the settings shown in the table.

The upright sitting, Fowler’s position, semi-Fowler’s position, and supine postures were accomplished using a Skytron Elite 6001 surgical table (Skytron LLC—Grand Rapids, MI, USA) available in the mock operating room environment of the Vanderbilt Institute of Surgery and Engineering (VISE). The surgical table replicates the experience of patients in healthcare settings as they recline from a completely upright position to a completely supine position. We determined participant posture based on the angle of reclination, which was manually adjusted by the investigator in accordance with Figure 4. Two bars attached to the side of the table were utilized to assist in determining the angle of reclination: one bar on the seat of the table that ran parallel to the surface of the floor, and one bar that ran parallel to the surface of the table that contacted the participant’s back. The angle of reclination was determined via a protractor. The chair was deemed ready for use when the angle of reclination reached a measurement in accordance with the values in the figure. Similarly to the standing position, participants were asked to remain still and avoid any shifting of their weight or heads while on the surgical table.

### 2.5. Experimental Design

**Figure 5:**
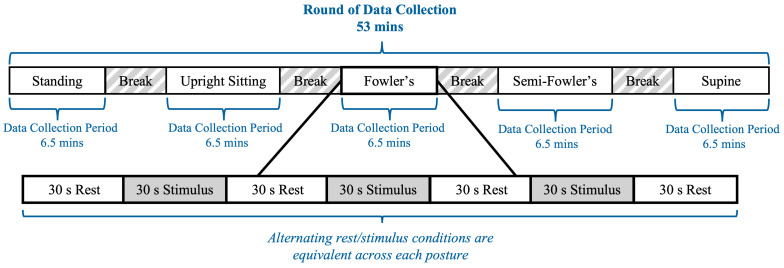
Timeline of the cw-fNIRS data collection period.

The experimental procedure was modeled after previous work by Shoushtarian et al., who investigated how different auditory stimulus intensities affected the heart rate of their seated participants as measured using fNIRS [25]. An overview of the experimental procedure is provided in Figure 5. The procedure included two rounds of data collection, with the second round being equivalent in arrangement and execution to the first; the rationale for a second round of data collection was to increase the overall data volume and enhance the reliability of the dataset. Each round contained five data collection periods lasting 6.5 min each; the 6.5 min periods consisted of alternating 30 s blocks of rest conditions and auditory stimuli, which followed block designs commonly used in fNIRS procedures. Each data collection period corresponded to one of the five postures, and the order of the positions during a given round was randomized for each participant. Between each data collection period, participants were granted five-minute breaks during which they assumed a standing position; the duration was selected to allow the researchers to adjust the setting of the surgical table. Considering all five data collection periods and breaks, the experimental procedure for one round was approximately 53 min. Between rounds, participants were granted a 10 min break during which they assumed a standing posture with the cw-fNIRS system still positioned on their heads.

The auditory stimuli consisted of three different intensities: 30 dB, 60 dB, and 90 dB, each randomized and delivered twice during an individual period and twenty times over the course of the procedure. The 30 dB, 60 dB, and 90 dB settings were chosen because they fall within the intensity range that has been used in previous fNIRS auditory studies [25] and, thus, provide a reference for interpreting the results of the study. Further, these auditory settings represent distinct intensity levels that span the typical safe range of human hearing: 30 dB is analogous to a whisper; 60 dB is analogous to a typical conversation; 90 dB is analogous to operating a hair dryer. The order of auditory stimulus intensity was randomized via a custom-created MATLAB (vR2024b) script. The input to the script was the ICRA noise file in waveform audio file (.wav) format, and the output was the 6.5 min audio script that could be played continuously for the participant. The auditory stimulus intensity was controlled through the MATLAB script and tested in advance via a decibel meter (TopTes—Hangzhou, China) to ensure the required intensity levels were achieved.

### 2.6. Data Analysis

The data were collected within the Oxysoft software (v3.4.12) that accompanies the Artinis Brite system and processed within the Homer3 Matlab fNIRS data processing toolbox [41]. The processing steps utilized for data analysis follow the hierarchy previously utilized by Chen et al. in their posture-focused study [9]. Raw data were captured as raw intensity values and converted to changes in optical density data, as is standard within cw-fNIRS processing workflows. The optical density data were then investigated channel-wise for sufficient signal quality, with bad channels (SNR < 20 dB) being pruned. Principal component analysis was then applied to the data along with motion-artifact removal (utilizing data from two 12 mm short channels) and bandpass filtering with cutoffs of 0.01 and 0.2 Hz to isolate the hemodynamic data from other physiological artifacts (e.g., Mayer waves, heart rate, respiration) [1,42]. The bandpass-filtered optical density data were then converted to changes in HbO and HbR concentrations using the modified Beer–Lambert law, assuming a source-detector separation distance of 30 mm for long channels and a differential pathlength factor calculated based on the participant’s age. We then performed block averaging for all 20 long-separation channels across the two rounds of data collection at each posture and compared the results.

For the stimulus component, we isolated the channel-wise HRF amplitude, which is defined as the difference between the mean HbO and HbR signal during the 30 s stimulus periods, for each posture. We then organized our HRF amplitude data into regional and global (meaning across all 20 long-separation channels in our layout) sets for comparison across the different postural conditions and auditory stimulus intensity settings.

For the resting component, we calculated the resting-state global amplitude as the standard deviation of the HbO time series across all 20 long-separation channels during the resting-state condition (no stimulus provided). We then performed power spectral density analysis on the resting-state global amplitude data using Welch’s method to assess low-frequency hemodynamic oscillations. Examining these low-frequency oscillations is helpful to understand the baseline signal components, which are more likely to be impacted by posture and less impacted by higher-frequency physiological noise. Based on the results of the power spectral density analysis, we performed frequency binning, which was also previously used by Chen et al. [9], to further analyze these baseline components.

We performed statistical analysis on both the task-induced HRF amplitude and resting-state global amplitude data. We grouped each of these datasets in two different ways: (1) for comparison across the five different postural conditions and (2) for comparison across the three different auditory intensities utilized. This testing was conducted using one-way repeated-measures ANOVA variance testing and post hoc Tukey’s Honestly Significant Difference (HSD) test to individually assess each condition for statistically different means while controlling for the family-wise error rate [43,44].

## 3. Results

### 3.1. Stimulus Condition Results

In order to validate our collected data and signal quality, we extended our analysis beyond the resting-state global amplitude, or the signal averaged across all 20 long channels, and investigated the HRF amplitudes for all participants (*n* = 15) across different functional regions of the brain. Our expectation was that regions implicated in speech comprehension would show a stronger hemodynamic response compared to other brain regions and that the recovered trends would match expected behavior based on known physiology. We separated the imaging channels into three groups based on their location and known brain function in speech comprehension. The first group encompasses the six most rostral channels in the imaging layout (left hemisphere: T7-FT7, FC5-FT7, FC5-C5; right hemisphere: FC6-FT8, T8-FT8, FC6-T6). These channels cover the inferior frontal gyrus (IFG), which generally functions in speech production, auditory comprehension, and executive function tasks such as action control and social cognition. While involved in speech and auditory comprehension, the major function of the IFG, which contains Broca’s area, is speech production and articulation. The second group encompasses the six most caudal channels in the layout (left hemisphere: P7-TP7, CP5-TP7, CP5-P5; right hemisphere: CP6-P6, P8-P6, P8-TP8), which cover the inferior temporal gyrus (ITG). The main function of the ITG is visual perception, visual processing, and object recognition, and it plays a minor role in speech comprehension. The final group encompasses the remaining eight channels (left hemisphere: T7-C5, T7-TP7, CP5-TP7, CP5-T5; right hemisphere: T8-C6, T8-TP8, CP6-TP8, CP6-C6) located between the IFG and ITG and covering the superior temporal gyrus (STG). The STG, which contains Wernicke’s area, primarily functions in speech comprehension, auditory processing, and the conversion of speech and audition into short-term memory.

**Figure 6:**
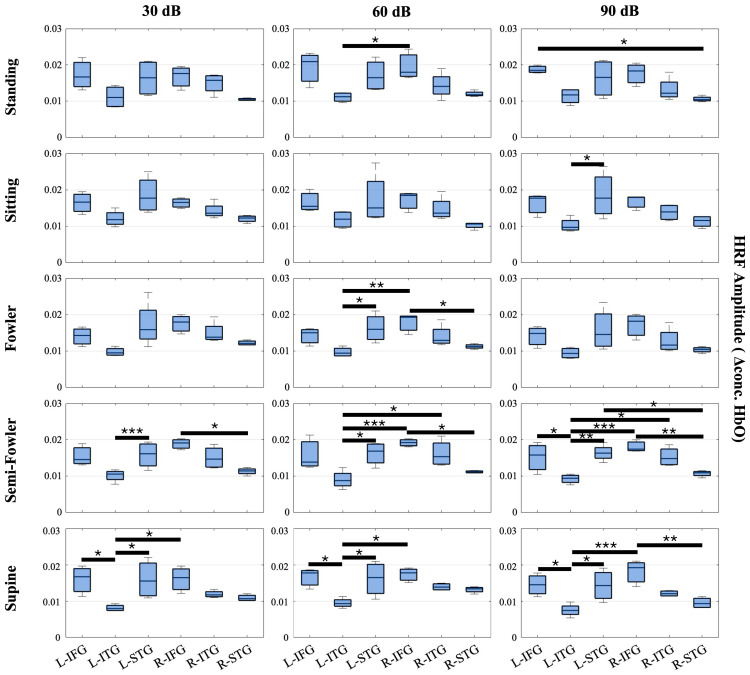
Box-and-whisker plots showing HRF signal amplitude for all postures and auditory stimulus settings across the channels covering the L-FIG, L-STG, L-ITG, R-FIG, R-STG, and R-ITG. These data represent the entire study population (*n* = 15 participants). Statistically significant relationships (* *p* < 0.05, ** *p* < 0.01, *** *p* < 0.005) from one-way repeated measures ANOVA and post hoc Tukey’s HSD are denoted on the plots.

Across these three groupings, we expected to see a strong hemodynamic response in the Group 1—IFG and Group 3—STG, as these functional regions specialize in auditory comprehension. The ITG falls secondary in these groupings as it is more visually based. Because we imaged bilaterally, we further divided the groups into the left and right hemispheres. We expected to see a greater response (i.e., higher HRF amplitude) in the left hemisphere of each region in comparison to the right because speech and language-based auditory stimuli are dominantly perceived in the left hemisphere. Figure 6 shows the results.

From this analysis, we found numerous statistically significant relationships (*p* < 0.05) when applying the one-way repeated measures ANOVA and post hoc Tukey’s HSD test. Amongst the relationships, 12 resulted from the L-STG or L-IFG regions producing a significantly higher HRF amplitude on average than the L-ITG. Additionally, seven relationships resulted from the R-IFG producing a significantly higher HRF amplitude than the L-ITG. While inter-hemispheric, this finding, along with the 12 intra-hemispheric relationships, is expected as both the left and right STGs and IFGs are strongly associated with speech comprehension, whereas the left and right ITGs are associated with visual processing. Further, the data considered were collected under the auditory task condition, wherein participants were masked to avoid external stimuli.

Amongst the other significant relationships, five resulted from the R-IFG producing a significantly higher HRF than R-STG. This finding was initially unexpected, considering the STG is the primary center for speech comprehension as previously stated; however, previous literature has shown that the R-IFG is activated in circumstances where speech is degraded or ambiguous [45,46]. We interpret that the nature of the ICRA noise, which has speech-like properties but is not decipherable as speech, led to the activation of the R-IFG that produced multiple statistically significant relationships and an average HRF amplitude that was, comparatively, one of the highest amongst the measured groups across posture and auditory intensity settings.

We observed more instances of significant relationships in the more reclined postures (semi-Fowler’s and supine) compared to more upright postures (standing, sitting, and Fowler’s) and an increasing number of significant relationships as the auditory stimulus intensity increased; however, statistically significant relationships were generally found across all measured postures and across all three auditory intensity settings. Overall, these significant relationships reinforce the quality of the collected data as the results are clearly interpretable and show expected trends when considering the functional regions, type of stimulus supplied, and previous literature.

**Figure 7:**
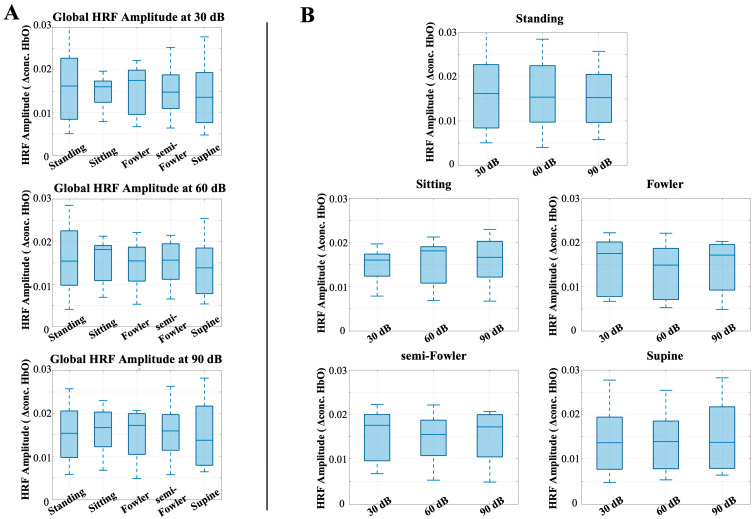
Box-and-whisker plots showing the global HRF amplitude for all postures across (**A**) 30 dB, 60 dB, and 90 dB auditory stimulus settings and across (**B**) each tested posture. One-way repeated-measures ANOVA was applied, but no relationships were found to be statistically significant.

To better understand the effect of posture on stimulus response, we next analyzed the global HRF amplitude for postural effects. Previous works have shown that the task-induced hemodynamic signal in response to auditory stimuli does not change with body position; however, we considered and analyzed these data to ensure this conclusion holds under Fowler’s and semi-Fowler’s postures. Figure 7 shows the results of this analysis, with each plot representing the global HRF amplitude for the labeled auditory intensity setting, grouped by intensity level (Figure 7A) or by posture condition (Figure 7B).

The one-way repeated-measures ANOVA test revealed no statistically significant differences across either posture or auditory intensity. This result is consistent with previous results obtained by Chen et al. [9], who also found no statistically significant difference across postures for the auditory stimulus condition and used the task-induced data segments as their control for the study. Our results reinforce that even with the introduction of reclined postures such as Fowler’s and semi-Fowler’s, the task-induced data are not significantly impacted when considering an auditory task.

### 3.2. Resting-State Results

**Figure 8:**
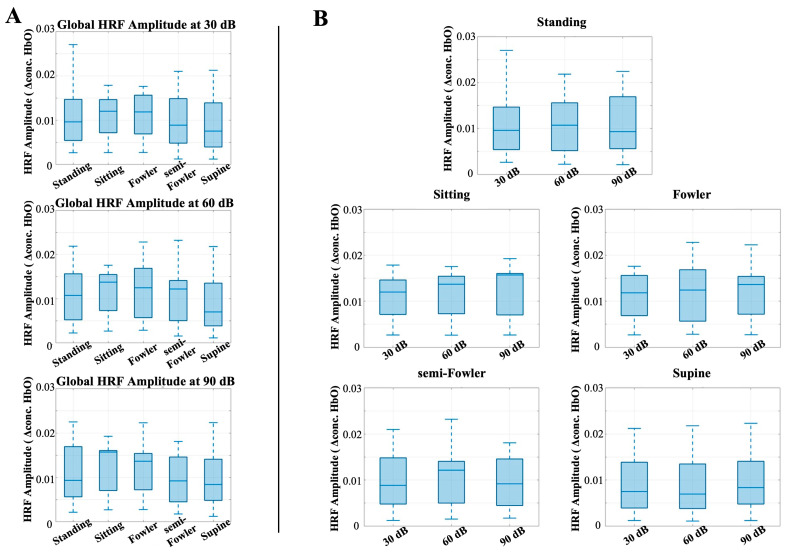
Box-and-whisker plots showing the global resting-state amplitude for all postures across (**A**) 30 dB, 60 dB, and 90 dB auditory stimulus settings and across (**B**) each tested posture. One-way repeated-measures ANOVA was applied, but no relationships were found to be statistically significant.

Similarly to the analysis for the auditory task condition, there were no statistically significant differences among the global resting-state amplitudes when applying the one-way repeated measures ANOVA analysis (Figure 8). These findings contradicted our initial hypothesis that the amplitude of the global resting signal would decrease as the angle of reclination decreased across postures.

**Figure 9:**
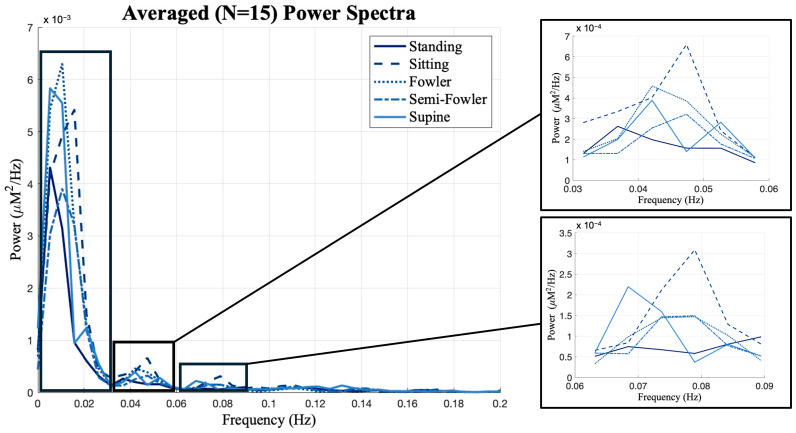
Global resting data results from power spectrum analysis using Welch’s method. The distinct peaks in the spectra led to frequency binning of data from 0 to 0.03, 0.03 to 0.06, and 0.06 to 0.09 Hz for further analysis. Box-and-whisker plots of the frequency-binned global resting data. Statistically significant relationships (* *p* < 0.05) from one-way repeated measures ANOVA and post hoc Tukey’s HSD are denoted on the plots.

Previous works from Chen [9] and Huo [22] showed that postures have unique impacts on data when observed in different frequency intervals. The global resting-state data consist of many different neural and physiological signals, and examining the frequency oscillations in the hemodynamic data is helpful to isolate the components that are most likely to be impacted by posture. Therefore, we investigated the power spectrum of the global resting-state data and found that the data had distinct peaks and differed most across postures at frequencies below 0.1 Hz. As shown in Figure 9, we performed frequency binning around these distinct peaks and grouped our global resting data into the following frequency bands: 0–0.03 Hz, 0.03–0.06 Hz, and 0.06–0.09 Hz. We then applied one-way ANOVA and post hoc Tukey’s HSD test across postures for each of these bands.

**Figure 10:**
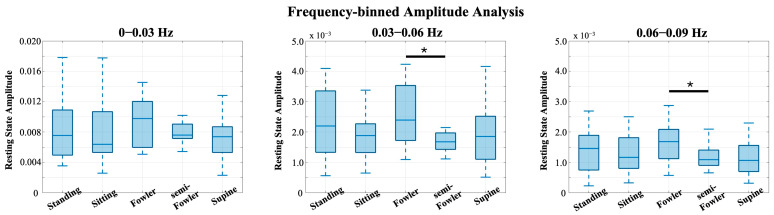
Box-and-whisker plots of the frequency-binned global resting data. Statistically significant relationships (* *p* < 0.05) from one-way repeated-measures ANOVA and post hoc Tukey’s HSD test are denoted on the plots.

From Figure 9, we see the global resting-state signal is dominantly less than 0.03 Hz with a peak component in our first bin. Specifically, this peak component is found around 0.02 Hz and was similarly found in the power analysis performed and reported by Chen [9]. In the statistical analysis performed across this bin, we found no statistically relevant impact of posture on signal (Figure 10).

We did, however, find statistically significant differences between Fowler’s and semi-Fowler’s postures in both of the 0.03–0.06 Hz and 0.06–0.09 Hz bins. In both bins, the amplitude of the global resting-state data for Fowler’s posture was significantly higher than that of semi-Fowler’s. Huo explains that very low frequency hemodynamic oscillations (~0.052 Hz) correspond to neurogenic or nerve-initiated activity [12]. These neurogenic activities are influenced by gravity and lead to changes in sympathetic activity and cortical arousal; thus, it is evident why a more upright posture, such as Fowler’s (comparatively more sympathetic activity and physiological arousal), would produce higher variability in signal and higher amplitude than the more reclined semi-Fowler’s in comparison. Further, work by Chen in this overlapping frequency window (f > 0.05 Hz) also showed this to be true, as they reported significantly higher amplitude for both standing and sitting postures in comparison to supine. Therefore, the statistically significant difference we observed between Fowler’s and semi-Fowler’s postures in these frequency windows partially supports our initial hypothesis, grounded in Chen’s results, that larger amplitudes would be found at higher angles of reclination. Our results do not fully support our initial hypothesis, as we did not find any statistically relevant relationships between standing, upright sitting, or supine postures. However, the significant relationship we observed between Fowler’s and semi-Fowler’s postures in these frequency windows represents an innovative finding, as to our knowledge, no other works with cw-fNIRS have performed data collection and analysis for these reclined postures.

We performed post hoc power analyses using G*Power (v4) [47] to properly interpret our results from the performed statistical analyses. Using our sample size (*n* = 15), alpha error (α = 0.05), empirically estimated within-subject correlations, and observed effect sizes, we observed power that ranged from 0.41 to 0.91. The powers calculated for the postural effects in the 0.03–0.06 and 0.06–0.09 Hz bins were 0.91 (Cohen’s f = 0.335) and 0.75 (Cohen’s f = 0.279), respectively. While the results from these specific analyses are moderately to sufficiently powered, the overall range for our analyses dips into being underpowered, and we recommend future studies with larger subject populations to more fully examine and reproduce these results.

## 4. Discussion

We tested the effect of five postures on the global resting signal and amplitude of the hemodynamic response function captured via cw-fNIRS and produced when patients were provided an auditory stimulus. The results from this study contextualized and distinguished the previously unknown effects of reclined postures, specifically Fowler’s and semi-Fowler’s, on cw-fNIRS hemodynamic data. In examining the amplitude of the HRF produced under auditory stimulus, our study expanded on the results from previous postural studies. Specifically, we showed that, similar to standing, upright sitting, and supine posture results previously reported by Chen et al. [9], reclined postures such as Fowler’s and semi-Fowler’s do not influence the task-induced response.

Further, we found that the low-frequency global resting data (f < 0.09 Hz) contained statistically relevant differences between Fowler’s and semi-Fowler’s postures, where Fowler’s produced significantly higher amplitude in comparison; this marks a novel finding of our work for cw-fNIRS. As mentioned previously in this manuscript, the study and comparison of Fowler’s and semi-Fowler’s postures is crucial as they represent two distinct and commonly assumed postures in clinical settings and, thus, require investigation to enable accurate and reliable data collection with cw-fNIRS systems in healthcare-relevant studies. This novel finding allows us to better distinguish the resting-state data across these more reclined postures while maintaining results that fit the general relationship between amplitude and posture previously reported by Chen [9] in the frequency range studied previously by Huo [22].

As a result of these findings, we do not recommend or foresee the need for more complex data processing pipelines or data segmentation efforts to resolve postural effects on the task-induced condition under auditory stimulus or on the general analysis of the global resting signal. However, researchers interested in the components of the resting-state signal, specifically components below 0.09 Hz, should consider the impacts of posture. We show that not all reclined postures are equivalent and would recommend that, for improved reliability and data context, researchers seek to report the angle of reclination measurements for seated data collection sessions.

Our work marks the first study of postural effects that considers Fowler’s, semi-Fowler’s posture conditions on cw-fNIRS data. These two postures are commonly assumed in healthcare settings and have yet to be studied in fNIRS literature; therefore, investigating the effects of these postures on hemodynamic data is a vital first step towards the accurate deployment of fNIRS systems in real-world environments where these postures are common, including in healthcare settings.

The study is limited by the relatively small sample size and the representative age of participants. While leveraging the data from 15 young (mean age 28.5 yrs), healthy participants enabled quality data for comparison of the HRF and global resting data across both posture and auditory stimulus intensity, our post hoc power analysis results suggest increasing the sample size by 30–40 participants in future work [48]. The initial study size was determined by averaging the population size of previously published postural studies using fNIRS that are referenced in this work; unfortunately, it is likely that other studies were possibly similarly underpowered, though their results do not specify this. Additionally, the clinical relevance of the study would improve with greater representation from older participants who better reflect clinical populations.

We also recommend further studies to investigate postural effects across different stimulus conditions to improve generalizability. Although probing auditory stimuli across the frontal and temporal gyrus has been performed in the past and is considered reliable on the group level, broader studies covering other sensory areas, possibly across the whole cortex, could provide much-needed insight if conducted under a similar paradigm as the present study.

We further suggest that follow-up studies, whether performed by our lab or other fNIRS researchers, across the auditory cortex additionally report each subject’s dominant ear, as is performed in dichotic listening studies [49]. While we did not outline this type of data to be collected in our IRB, and thus do not report it, it could supplement the hemispheric analysis in future studies.

Further, future studies attempting to broaden the generalizability of this work, whether by broadening coverage or using non-auditory stimuli, should also look to complement resting-state hemodynamic data with physiological measurements such as heart rate or respiration. The resting-state analysis we performed would have been enriched by this additional data had we thought to include this data collection in our initial study planning.

Our study provides an improved understanding of the effects of postures commonly assumed in healthcare settings, such as supine, Fowler’s, and semi-Fowler’s, on the hemodynamic measurements made with cw-fNIRS systems during resting-state conditions. Specifically, the amplitude of the global resting-state signal differs significantly between Fowler’s and semi-Fowler’s postures at frequencies below 0.09 Hz; therefore, researchers interested in studying the resting state while participants assume a sitting posture should at least note, if not report, the angle of reclination. These novel insights can be used to improve the accuracy of neuroimaging results collected in healthcare settings and other settings outside of the typical lab environment.

## Data Availability

The data presented in this study may be available on request from the corresponding author due to ethical considerations and Institutional Review Board approval.

## Abbreviations

The following abbreviations are used in this manuscript:

fNIRS	Functional Near-Infrared Spectroscopy
HRF	Hemodynamic Response Function
cw-fNIRS	Continuous Wave-Functional Near-Infrared Spectroscopy
TCD	Transcranial Doppler
MRI	Magnetic Resonance Imaging
HbO	Oxygenated Hemoglobin
HbR	Deoxygenated Hemoglobin
EEG	Electroencephalography
IRB	Institutional Review Board
ICRA	International Collegium of Rehabilitative Audiology
VISE	Vanderbilt Institute of Surgery and Engineering
SNR	Signal-to-Noise Ratio
ANOVA	Analysis of Variance
HSD	Honestly Significant Difference
IFG	Inferior Frontal Gyrus
ITG	Inferior Temporal Gyrus
STG	Superior Temporal Gyrus
